# Isotope tracing reveals distinct substrate preference in murine melanoma subtypes with differing anti-tumor immunity

**DOI:** 10.1186/s40170-022-00296-7

**Published:** 2022-12-01

**Authors:** Xinyi Zhang, Alexandra A. Halberstam, Wanling Zhu, Brooks P. Leitner, Durga Thakral, Marcus W. Bosenberg, Rachel J. Perry

**Affiliations:** 1 Department of Internal Medicine (Endocrinology), Yale School of Medicine, P.O. Box 208026, 333 Cedar St., SHM BE36-B, New Haven, CT 06520-8026 USA; 2 Department of Cellular & Molecular Physiology, Yale School of Medicine, New Haven, USA; 3 Department of Pathology, Yale School of Medicine, New Haven, USA; 4 Department of Dermatology, Yale School of Medicine, New Haven, USA; 5 Department of Immunobiology, Yale School of Medicine, New Haven, CT USA; 6 Yale Stem Cell Center, New Haven, CT USA; 7 Yale Cancer Center, New Haven, CT USA; 8 Yale Center for Immuno-Oncology, New Haven, CT USA

**Keywords:** Melanoma, Tumor metabolism, Tumor microenvironment, Glucose, Amino acid

## Abstract

**Background:**

Research about tumor “metabolic flexibility”—the ability of cells to toggle between preferred nutrients depending on the metabolic context—has largely focused on obesity-associated cancers. However, increasing evidence for a key role for nutrient competition in the tumor microenvironment, as well as for substrate regulation of immune function, suggests that substrate metabolism deserves reconsideration in immunogenic tumors that are not strongly associated with obesity.

**Methods:**

We compare two murine models: immunologically cold YUMM1.7 and immunologically-hot YUMMER1.7. We utilize stable isotope and radioisotope tracer-based metabolic flux studies as well as gas and liquid chromatography-based metabolomics analyses to comprehensively probe substrate preference in YUMM1.7 and YUMMER1.7 cells, with a subset of studies on the impact of available metabolites across a panel of five additional melanoma cell lines. We analyze bulk RNA-seq data and identify increased expression of amino acid and glucose metabolism genes in YUMMER1.7. Finally, we analyze melanoma patient RNA-seq data to identify potential prognostic predictors rooted in metabolism.

**Results:**

We demonstrate using stable isotope tracer-based metabolic flux studies as well as gas and liquid chromatography-based metabolomics that immunologically-hot melanoma utilizes more glutamine than immunologically-cold melanoma in vivo and in vitro. Analyses of human melanoma RNA-seq data demonstrate that glutamine transporter and other anaplerotic gene expression positively correlates with lymphocyte infiltration and function.

**Conclusions:**

Here, we highlight the importance of understanding metabolism in non-obesity-associated cancers, such as melanoma. This work advances the understanding of the correlation between metabolism and immunogenicity in the tumor microenvironment and provides evidence supporting metabolic gene expression as potential prognostic factors of melanoma progression and may inform investigations of adjunctive metabolic therapy in melanoma.

**Trial registration:**

Deidentified data from The Cancer Genome Atlas were analyzed.

**Supplementary Information:**

The online version contains supplementary material available at 10.1186/s40170-022-00296-7.

## Background

Melanoma is the most lethal form of skin cancer [1]. Checkpoint-blockade immunotherapy has significantly decreased mortality from advanced-stage melanoma. Long-term outcomes in melanoma patients treated with checkpoint inhibitors are limited by the development of immunotherapy resistance, especially in late-stage melanoma patients with metastasis. This limitation means that there is an urgent need to develop better (or adjuvant) therapies for melanoma treatment. Metabolic therapies, which may decrease tumor cell fitness relative to healthy cells, could hold therapeutic promise in this area. Our study begins to investigate this possibility by seeking to identify which tumor type—those that are responsive to immunotherapy (“immune hot”) and those that are not (“immune cold”)—might respond best to metabolic therapies.

For more than a century, researchers have recognized metabolism as a mediator of tumor cell fitness. The Warburg effect describes tumor cells’ pathognomonic reliance on glycolysis to produce ATP and building blocks for rapid cell division [2–4]. Investigators have taken multiple approaches to exploit the Warburg effect. Some of these approaches have been effective preclinically. For example, dichloroacetate (DCA) inhibits pyruvate dehydrogenase kinase, activating pyruvate dehydrogenase and driving pyruvate into the tricarboxylic acid (TCA) cycle [5]. In vitro DCA treatment increases apoptosis, slows tumor cell division, and increases chemotherapy efficacy [6–9], likely by diverting substrates away from biosynthetic pathways required for cell division.

Recent work has also highlighted the importance of metabolism in immune cell function. During the anti-tumor immune response, tumor-infiltrating lymphocytes (TILs) undergo a metabolic switch. Following activation, TILs change their primary energy source from oxidative phosphorylation to glycolysis to meet increased energy requirements and generate necessary material for cell division and differentiation [10, 11]. In short, high-functioning TILs rely on glycolysis. Therefore, researchers have sought to improve responsiveness to immunotherapy by modulating glycolysis using small molecules [12–16]. Given their expected impact to disturb normal metabolic processes in vivo, however, the translational potential of such systemic metabolic strategies may be limited.

Thus, researchers have turned to pathways that may enhance T cell activation and/or impede exhaustion while exerting a less profound effect on metabolic homeostasis. In examinations of other immune cell types, researchers have described glutamine as the “fuel of the immune system” [17]. Lymphocytes increase the uptake of glutamine during the anti-tumor response. Researchers have also suggested that glutamine may be essential for lymphocyte proliferation and function [18]. Supplementing lymphocytes and macrophages with glutamine, for instance, enhances function [19, 20]. Recent studies, however, indicate that the direction of this relationship is unclear. For example, Nabe et al. recently showed that glutamine deprivation during in vitro activation improves T cell tumor infiltration and responsiveness to anti-PD1 treatment in tumor-bearing mice [21]. Most investigations into the role of glutamine have focused on the impact of glutamine metabolism in TILs in vitro, while largely ignoring the impact of glutamine on tumor cell division and function in the in vivo tumor microenvironment (TME). However, pharmacologic interventions to manipulate metabolism in vivo are difficult to administer in a cell-specific manner; most small molecules administered systemically will affect immune cell metabolism and, simultaneously, metabolism in tumor cells and other tissues. Our study seeks to combine in vivo and in vitro studies implanting [^13^C] tracer methodology combined with gas and liquid chromatography-based analysis to address these gaps.

In this report, we compare two common murine melanoma models: YUMM1.7 and YUMMER1.7 [22], adding five additional cell lines with a variety of immunogenicity for confirmatory studies. YUMM1.7 is “immunologically cold,” meaning that lymphocytes minimally infiltrate YUMM1.7 tumors and that YUMM1.7 does not respond to immune checkpoint treatments. YUMMER1.7 is derived from YUMM1.7, but in contrast to YUMM1.7, YUMMER1.7 is “immunologically hot.” YUMMER1.7 therefore has high T cell infiltration and responds to immune checkpoint treatments [22]. However, the interplay between metabolic changes and immunogenicity is not fully understood.

In this study, we demonstrate an association between immune “hot” or “cold” status and substrate utilization in the TME by comparing gene expression, metabolic flux, and cell survival under nutrient-depleted conditions in two murine melanoma models, with confirmatory studies in five more. We demonstrate that glutamine transporter expression is positively correlated with lymphocyte infiltration and anti-tumor effector function. This work advances the understanding of the relationship between tumor substrate preference/utilization and tumor cell division in the TME. This work may also influence the design of clinical trials investigating metabolic adjuvants to immunotherapy in melanoma, with a particular emphasis on targeting tumor metabolism to, in turn, optimize the ability of the immune system to fight melanoma.

## Methods

### Cells

We purchased YUMM1.7, B2905, and B16-F10 cells from ATCC (CRL-3362, Cellosaurus Accession Number CVCL_JK16; and CRL-3476, Cellosaurus Accession Number CVCL_B0CG; and CRL-6475, Cellosaurus Accession Number CVCL_0159, respectively) and D4M-3A cells from Sigma (SCC428, Cellosaurus Accession Number CVCL_0P27) and obtained YUMMER1.7 cells from the Bosenberg laboratory, where they are regularly authenticated. We did not authenticate the cells purchased commercially, because this is routinely done by the suppliers. We maintained cells from both lines in the media shown in Table 1.

**Table 1:** Media used for maintenance of cell lines

Cell line	Base media	Supplements
YUMM1.7	DMEM-F12	10% FBS, 2.5 mM glutamine, 0.5 mM sodium pyruvate, 1200 mg/L sodium bicarbonate, non-essential amino acids
YUMMER1.7	DMEM-F12	10% FBS, 2.5 mM glutamine, 0.5 mM sodium pyruvate, 1.2 g/L sodium bicarbonate, non-essential amino acids
B2905	RPMI-1640	10% FBS, 10 g/L glutamine
B16-F10	DMEM	10% FBS
D4M-3A	RPMI-1640	10% FBS, 2.0 mM glutamine

In all cases, the FBS was not dialyzed, and 1% penicillin/streptomycin was also included. For the irradiation studies, B2905 and D4M-3A were thrice UVB irradiated (600 J/m^2^) [23] within 24 h prior to injection. In all cases, we injected cells into mice after passage <10. In in vitro flux studies, we serum-starved cells for 14 h prior to incubation in a tracer. We supplied tracer media containing [^13^C_6_] glucose (5 mM), [^13^C] palmitate/oleate (2.5 mM of each) or [^13^C_5_] glutamine (5 mM). We also provided unlabeled substrates at these concentrations to ensure that substrates were not limiting, regardless of ^13^C label (in other words, in cell survival experiments and all other studies, “full media” refers to 5 mM glucose/5 mM fatty acid (1:1 palmitate to oleate)/5 mM glutamine). We collected cells in 50% ethanol after the incubation times indicated in the figures. For cell survival experiments, we plated cells in recommended media in 24-well plates, with three wells per condition (i.e., media containing glucose and glutamine, but no palmitate; media containing glucose and palmitate, but no glutamine, etc.). At 24 h, 48 h, and 72 h after plating, cells are stained with Trypan blue and counted (3 measurements per condition) by a LUNA-II automated cell counter.

Lactate production was measured in cells incubated in lactate-free but otherwise complete media for 6 h. The rate of lactate production was assumed to be linear over the 6-h period and was normalized to total protein content measured using the BCA protein assay.

### Mitochondrial stress test

To examine mitochondrial and non-mitochondrial respiration, we performed a mitochondrial stress test using the Seahorse XF Flux Analyzer. Studies were performed with cells at 70–80% confluency, and data normalized to total protein. The protocol used for the mitochondrial stress test has been published previously [24].

### Mice

The Yale Institutional Animal Care and Use Committee approved all animal studies. We purchased wild-type male C57bl/6J mice from Jackson Laboratories (Bar Harbor, ME) at 8 weeks of age. After 1 week of acclimation, we injected mice subcutaneously in the right chest with 5x10^5^ YUMM1.7 or YUMMER1.7 tumor cells after confirmation that they were pathogen-free (including mycoplasma) by the Yale Comparative Pathology Research Core. Three weeks after tumor cell injection, we performed surgery under isoflurane anesthesia to implant catheters in the jugular vein for tracer infusion (described in the “Flux studies” section). Tumor interstitial fluid was isolated from a separate set of mice not infused with tracer. Tumors were minced in PBS (2:1 v/w PBS to tumor), transferred to a centrifuge tube, and 8:1 PBS to tumor weight added. Samples were then incubated at 37°C for 60 min, centrifuged at 2000 rpm for 10 min, and the supernatant (tumor interstitial fluid) was collected.

### Biochemical analysis

We measured glucose and lactate concentrations enzymatically using the YSI 2500 Glucose/Lactate Analyzer (Yellow Springs, OH, USA). We measured glutamine concentration and enrichment by LC-MS/MS (AbSCIEX 6500 QTRAP with a Shimadzu ultrafast liquid chromatography system in negative ion mode) as we have previously reported [25] after spiking with [^2^H_4_] taurine as an internal standard. We measured citrate, succinate, and malate concentration and enrichment using the same LC-MS/MS method. Mass/charge ratios for the unlabeled metabolites are shown in Table 2. Due to space constraints, the labeled intermediate mass/charge ratios are not shown.

**Table 2:** Mass/charge ratios for the unlabeled metabolites

Analyte	Mass/charge
Citrate	191/173
Glutamate	146/128
Malate	133/115
Succinate	117/99

We measured amino acid concentrations with gas chromatography/mass spectrometry after derivatization as described previously [26]. We measured non-esterified fatty acid (NEFA) concentrations using the Wako NEFA-HR assay (Mountain View, CA, USA).

### Flux studies

Mice underwent a primed (3X)-continuous infusion of [U-^13^C_5_] glutamine, [U-^13^C_6_] glucose, or [U-^13^C_16_] palmitate (continuous infusion rate for each tracer, all 1 mg/kg body weight/min), or [^14^C] glutamine, [^3^H] glucose, or [^14^C] palmitate (continuous infusion rate 1 μCi/kg/min) for 60 min, unless otherwise stated. All stable isotopes were obtained from Cambridge Isotopes (Tewksbury, MA, USA), and all radioisotopes from PerkinElmer (Waltham, MA, USA). Within 30 s of euthanizing animals with IV pentobarbital (Covetrus; Portland, ME, USA), we isolated and freeze-clamped tumors in liquid nitrogen.

In method-developing trials, we observed that regardless of tracer identity ([^13^C] glutamine, glucose, or palmitate), accumulation of [^13^C] label was linear between 0 and 30 min and by comparing citrate enrichment in tumors of mice infused for 60 and 150 min, ascertained that 60 min of infusion was sufficient to reach steady-state under these conditions. Therefore, we infused mice with [^13^C] isotope for 60 min in the steady-state experiments. We calculated relative V_CS_ as the slope of [^13^C] citrate enrichment measured at 60 min vs. time and normalized to YUMM1.7 tumors infused with [^13^C_6_] glucose. In mice infused with [U-^13^C_6_] glucose, the ratio of [^13^C_3_] pyruvate/[^13^C_6_] glucose was used as a readout of glycolysis, although we acknowledge that this ratio is not a direct readout of glycolysis. In in vivo radioisotope infusion studies, we assumed that [^14^C] or [^3^H] label would reach a steady state at a similar timepoint as [^13^C] label and thus terminated radioisotope infusion studies at 60 min.

### Flow cytometry

We measured in vitro cell viability and PD-L1 expression using the BD LSR II flow cytometer. Cells were digested into single cells with trypsin EDTA (0.25%), followed by cell viability staining (ThermoFisher Scientific, #L34964) for 20 min at room temperature. After washing with PBS two times, cells were either ready for viability analysis or stained with PD-L1 antibody (ThermoFisher, #12-5982-82) for 20 min under room temperature and washed with PBS three times. In both cases, cells were fixed by 4% polymeric formaldehyde (PFA) after staining and analyzed within 24 h.

### RNA-seq analysis

All RNA-seq analysis follows standard DESeq workflow. YUMM1.7 and YUMMER1.7 bulk RNA-Seq data are available upon request from author Marcus W. Bosenberg and the remainder of the data from corresponding author Rachel J. Perry. The human PRECOG database, a meta-analysis of gene expression, and overall survival data are available at https://precog.stanford.edu/, and the TCGA human tumor gene expression data (TCGA Melanoma (SKCM)) can be downloaded pre-filtered and normalized at https://xenabrowser.net/datapages/.

The principal component analysis, volcano plot, and correlation maps were generated in R. Statistical significance for genes revealed by differential expression analysis was defined as a Log_2_(Fold Change) >2 or ≤2 and a Benjamini-Hochberg corrected *p* value of <0.01. PRECOG datasets were downloaded, and the melanoma and metastatic melanoma meta-analyses were filtered to metabolic gene sets downloaded from the Kyoto Encyclopedia of Genes and Genomes (KEGG). *Z* scores in the PRECOG dataset reflect the effect of high gene expression on overall survival in publicly available tumor gene expression datasets, where a *Z* score > 3.09, which correlates to a *p* value 0.001, corresponds to a detrimental effect on survival, while a *Z* score < −3.09 corresponds to a beneficial effect on survival with a *p* value of 0.001.

The heatmap of gene expression was generated in Python, with an automated clustering algorithm was employed using Euclidean distances between genes, and with a *Z* score applied to each row, using Seaborn’s Clustermap function.

All code used to generate figures is available online (https://github.com/xz710/YUMM-YUMMER-comparison.git).

### Statistical analysis

We used the 2-tailed unpaired Student’s *t* test to compare 2 groups, and ANOVA with Tukey’s multiple comparisons test to compare 3 or more groups, in GraphPad Prism Version 9. In all cases, we verified that the data met the assumptions of the statistical test employed.

## Results

### YUMMER1.7 cells utilize more glucose and glutamine than YUMM1.7 cells in vitro

**Fig. 1:**
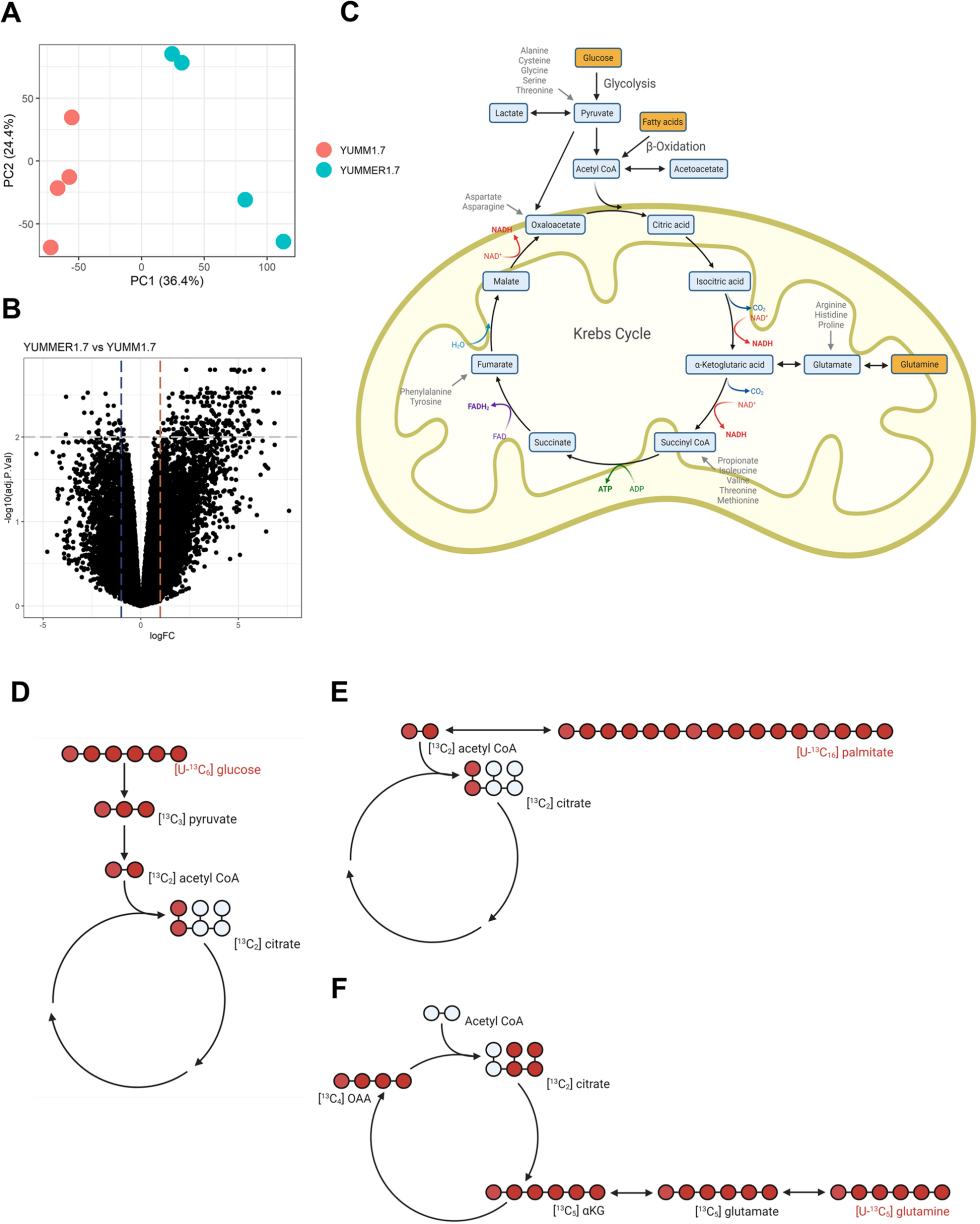
RNA sequencing and isotope tracers were used to study metabolic pathways. **A** Principal component analysis (PCA) of bulk RNA-seq in YUMM1.7 and YUMMER1.7 cell lines. **B** Volcano plot of differentially expressed gene comparing YUMMER1.7 and YUMM1.7 cell bulk RNA-seq, Log_2_(fold change)>2, *P*<0.01 define the dotted line cutoffs. **C** Schematic depicting mitochondrial metabolism. Metabolites traced in this study are shown in yellow boxes and anaplerotic substrates in gray text. Figures in panels **C**–**F** were created with Biorender.com. **D**–**F** Tracer-labeling scheme reflecting the first turn of the TCA cycle when [U-^13^C_6_] glucose, [U-^13^C_16_] palmitate, and [U-^13^C_5_] glutamine is used as a tracer, respectively

We first sought to identify differences between the RNA expression of metabolic genes in YUMM1.7 and YUMMER1.7 murine melanoma models. We hoped such differences might indicate a mechanism contributing to the immunogenic capacity of the tumor. To avoid confounding by the presence or absence of TILs, we compare bulk RNA sequencing data from YUMM1.7 and YUMMER1.7 cell lines. Principal component analysis (PCA) revealed distinct separation between the two cell lines, with 36.4% of the variance in the datasets explained by the first principal component, along which the samples appeared to separate (Fig. 1A). We identified 862 genes expressed differently between these models (Fig. 1B). Some of these genes were key metabolic regulators. For instance, *Slc43a1* and *Slc43a3* encode neutral amino acid transporters, *Cd36* encodes a glycoprotein functioning as a fatty acid transporter in many tissues, and *Gfpt2* encodes glutamine-fructose-6-phosphate transaminase 2, which controls glucose flux into hexosamine pathway (Supplementary Table S1). We then tested whether the observed differences in gene expression correlated to substrate flux through these enzymes.

To do so, we used stable isotope tracers to compare oxidation of glucose, glutamine, and fatty acids between YUMM1.7 and YUMMER1.7 cells (Fig. 1C–F). We chose these metabolites as they are the three most important energy resources in most cells. We calculated the TCA cycle flux driven by each nutrient by measuring the enrichment of ^13^C-labeled metabolites using gas and liquid chromatography-mass spectrometry (GC-MS and LC-MS/MS, respectively). We first confirmed that cells reached an isotopic steady state in all isotopologues ^13^C citrate after incubation in media containing either [U-^13^C_6_] glucose, [U-^13^C_5_] glutamine, or [U-^13^C_16_] palmitic acid within 30 min (Supplementary Figure S1A-S1R).

**Fig. 2:**
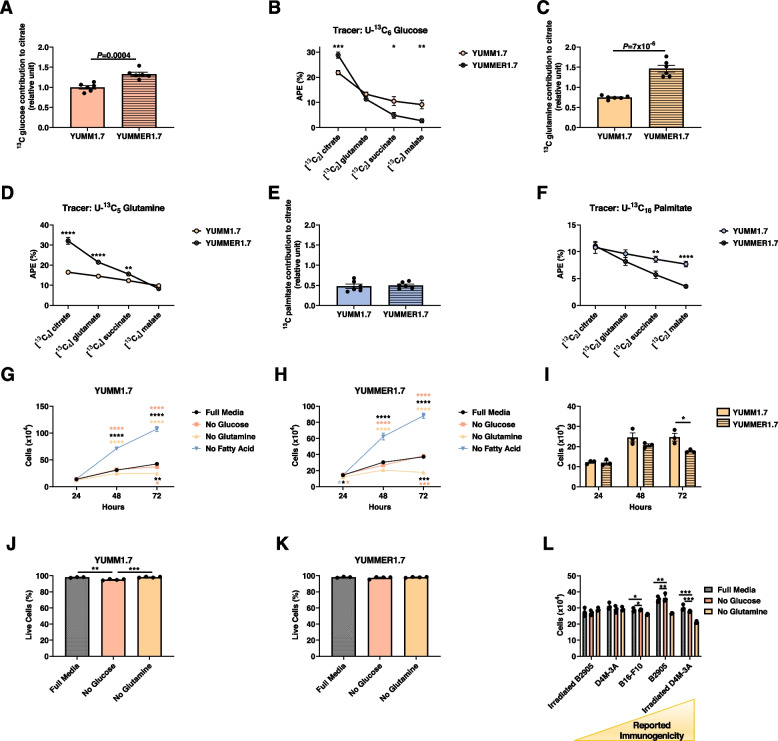
YUMMER1.7 cells are more reliant on anaplerotic substrates in vitro than YUMM1.7 cells. **A** V_CS_ from [U-^13^C_6_] glucose. **B** ^13^C enrichment of TCA cycle intermediates in cells incubated in [U-^13^C_6_] glucose. **C** Relative V_CS_ in cells incubated in [U-^13^C_16_] glutamine, normalized to data from cells incubated in [U-^13^C] glucose. **D** ^13^C enrichment of TCA cycle intermediates in cells incubated in [U-^13^C_6_] glutamine. **E** Relative V_CS_ in cells incubated in [U-^13^C_16_] palmitate. **F** ^13^C enrichment of TCA cycle intermediates in cells incubated in [U-^13^C_6_] glucose. **G**–**H** YUMM1.7 and YUMMER1.7 cell number under conditions of substrate deprivation in vitro. The color of the asterisks denotes the group to which the group closest to the symbols was compared. **I** YUMM1.7 and YUMMER1.7 cell number under conditions of glutamine deprivation. **J**, **K** Effect of glucose and glutamine deprivation on the fraction live/dead cells, assessed by flow cytometry. **L** Impact of glucose or glutamine deprivation on cell number in five cell lines with varying immunogenicity. In all panels, *P<0.05, **P<0.01, ***P<0.001, ****P<0.0001. The 2-tailed unpaired Student’s *t* test was used to compare two groups and ANOVA with Tukey’s multiple comparisons test to compare three or four groups

We measured [^13^C_2_] citrate derived from [^13^C_6_] glucose as a readout of the contribution of glucose to the TCA cycle. Relative citrate synthase flux (V_cs_), defined as the slope of [^13^C] citrate enrichment measured at 30 min, represents the metabolic flux entering the TCA cycle from glycolysis. We observed a higher contribution of glucose to citrate in YUMMER1.7 as compared to YUMM1.7 cells, indicating higher glucose oxidation in immune “hot” YUMMER1.7 cells (Fig. 2A). Moreover, we observed a higher dilution of [^13^C] glucose label throughout the TCA cycle in YUMMER1.7, indicating higher anaplerotic influx into the TCA cycle from other unlabeled substrates (Fig. 2B, Supplementary Figure S2A-E). Glutamine contribution to V_CS_ was also higher in YUMMER1.7 cells, as was the dilution of the label through the TCA cycle, reflecting anaplerosis (Fig. 2C, D and Supplementary Figure S2F-J). The contribution of fatty acids to the TCA cycle was similar between the two models, but anaplerosis, as expected, remained greater in YUMMER1.7 cells (Fig. 2E, F and Supplementary Figure S2F). These data were expected because rates of anaplerosis (i.e., unlabeled substrate entry into the TCA cycle) are unaffected by which tracer is used.

We also measured tumor cell proliferation while depriving cells of key nutrients in vitro. Our data demonstrate that glucose deprivation had no impact on either YUMM1.7 or YUMMER1.7 cell proliferation in vitro, whereas glutamine deprivation inhibited cell proliferation in both models (Fig. 2G, I). Moreover, incubation in free fatty acid had a deleterious effect in vitro, which further suggests that fatty acid is unlikely to promote melanoma anti-tumor immune cell function in the melanoma TME. This effect may be attributable in part to palmitate toxicity, which has been observed in multiple cell types including adipocytes [27], hepatocytes [28], and cardiomyocytes [29], among others; however, we anticipate that the inclusion of the unsaturated fatty acid oleate may mitigate this toxicity, as has been demonstrated recently in additional cell types [30, 31]. The fact that the tumor cell number curves are not exponential growth suggests that cells may be approaching a horizontal asymptote in the division at the 72-h time point. This effect is unlikely to result from substrate limitation considering the abundant, substrate-replete, media provided, but is more likely a result of approaching confluency and resulting physical changes. It is unlikely that these effects were the result of increased cell death with substrate deprivation, while glucose deprivation had a small (2.5%) effect to reduce the fraction of live YUMM1.7 cells, glutamine deprivation had no effect, and neither substrate affected the fraction of live YUMMER1.7 cells (Fig. 2J, K).

We then expanded the scope of our study of the impact of available substrates on melanoma cells by examining the impact of substrate depletion on five additional melanoma cell lines, with a range of immunogenicity: UVB-irradiated B2905 [23], D4M-3A [32, 33], B16-F10 [34], not irradiated B2905 [23], and irradiated D4M-3A. Our data reveal an increasing dependence upon glutamine in the more immunogenic cell lines: incubation in media lacking glutamine had a greater effect to suppress cell division in B2905 and irradiated D4M-3A cells, both of which are considered more immunogenic melanoma cell lines, as compared to the others (Fig. 2L).

**Fig. 3:**
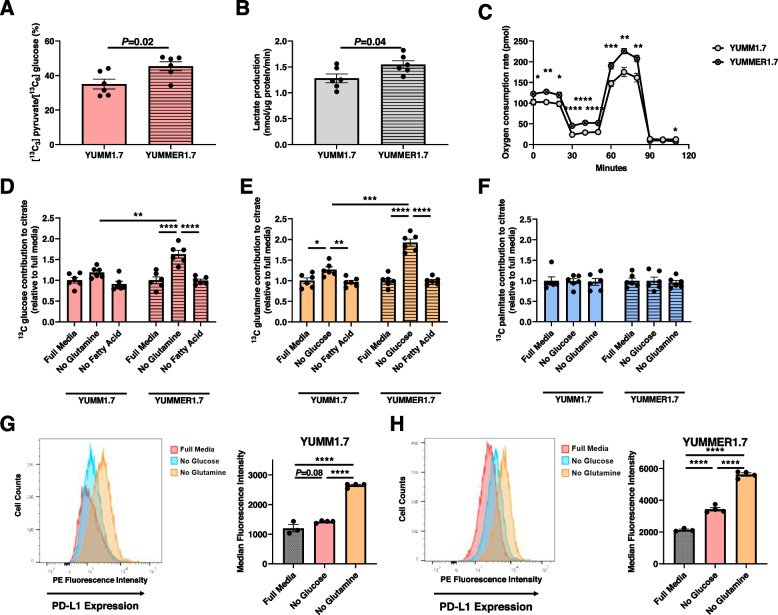
Glutamine and glucose deprivation increase utilization of the other substrate and increase tumor PD-L1 expression. **A** [^13^C_3_] pyruvate/[^13^C_6_] glucose in cells incubated in [U-^13^C_6_] glucose. **B** Lactate production by YUMM1.7 and YUMMER1.7 melanoma cells. **C** Oxygen consumption during a mitochondrial stress test. **D**–**F** Contribution of ^13^C glucose, glutamine, and palmitate to citrate in YUMM1.7 and YUMMER1.7 cells. **G**, **H** Impact of glucose and glutamine deprivation on YUMM1.7 and YUMMER1.7 PD-L1 expression, measured by flow cytometry. In all panels, comparisons within cell lines were performed by ANOVA with Tukey’s multiple comparisons test, and comparisons between cell lines by the 2-tailed unpaired Student’s *t* test. *P<0.05, **P<0.01, ****P<0.0001. a.u. arbitrary unit

Melanoma cells shift their metabolism, to varying effects, in response to substrate deprivation. YUMMER1.7 cells showed a greater reliance upon glucose as reflected by an increased conversion of ^13^C glucose to ^13^C pyruvate and increased production of lactate in vitro, both resulting from glycolysis (Fig. 3A, B) in addition to basal respiration, ATP production, and maximal respiration during a mitochondrial stress test (Fig. 3C). To directly understand how substrate deprivation affects tumor cell metabolism, we performed a series of in vitro studies incubating YUMM1.7 and YUMMER1.7 cells in glucose-, fatty acid-, and glutamine-deprived media. We observed that upon incubation in glucose- or glutamine-free media, cells increased their oxidation of the other substrate, whereas incubation in fatty acid-free media did not alter the oxidation of glucose or glutamine (Fig. 3D–F).

Glucose and glutamine deprivation increases tumor PD-L1 expression. Next, to generate a direct readout of the impact of tumor cell substrate metabolism on tumor immunogenicity, we incubated YUMM1.7 and YUMMER1.7 cells in media lacking glucose or glutamine for 48 h and used flow cytometry to demonstrate how these perturbations affected cancer cell PD-L1 expression. As expected, PD-L1 expression was lower in “immune cold” YUMM1.7 than in “immune hot” YUMMER1.7 (Fig. 3G, H). Glucose deprivation, and to a greater extent glutamine deprivation, increased PD-L1 expression in both cell lines. Checkpoint inhibitors may have greater potential efficacy in tumors with higher PD-L1 expression, so while these data do not prove the impact of substrate metabolism on immunogenicity in melanoma cells, they do directly link tumor cell substrate metabolism to immunogenicity status.

**Fig. 4:**
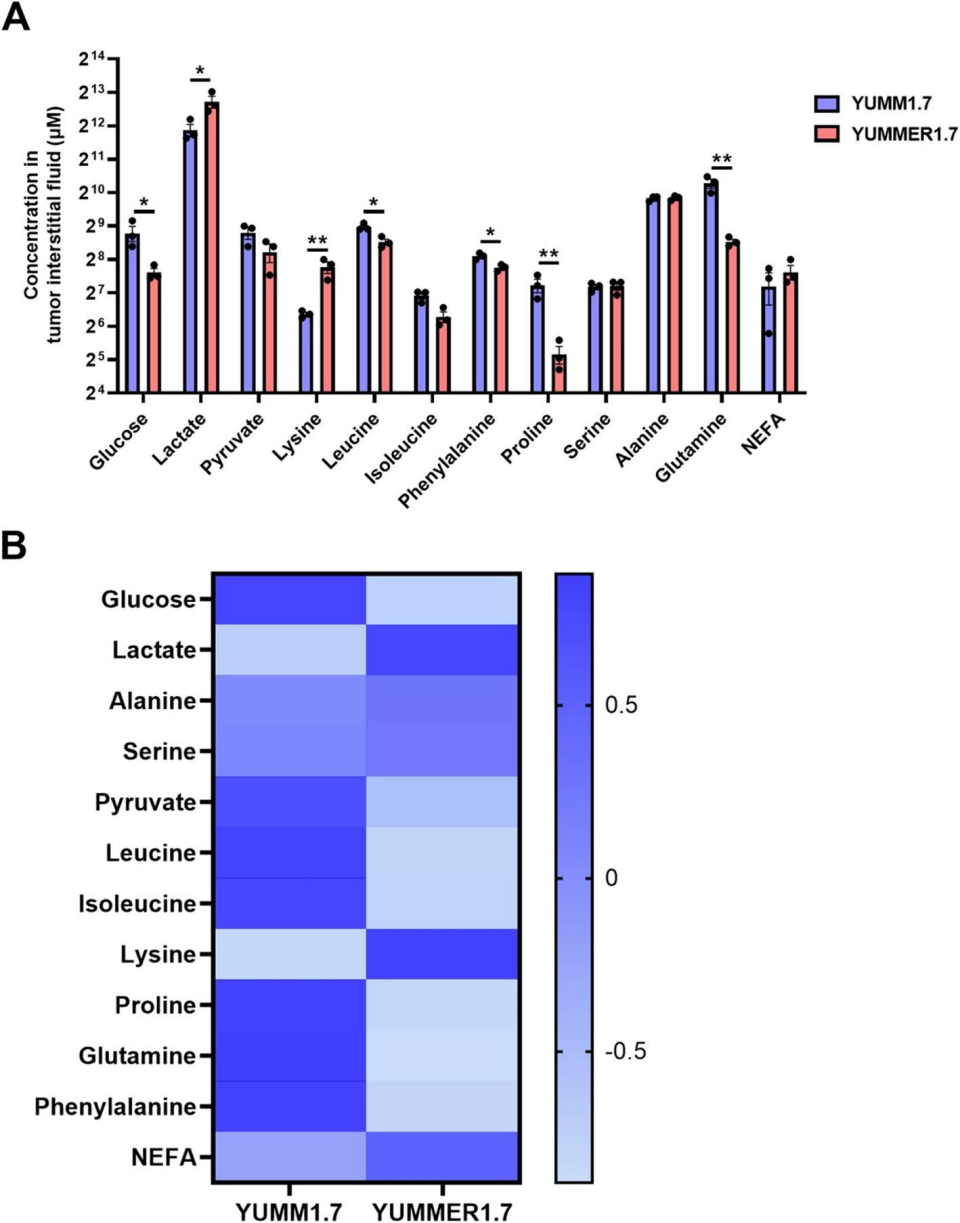
Analysis of tumor interstitial fluid metabolite concentrations ex vivo suggests greater utilization of glucose and amino acids in YUMMER1.7 tumors as compared to YUMM1.7. **A** Metabolite concentrations. *P<0.05, **P<0.01 by the 2-tailed unpaired Student’s *t* test. **B** Heatmap showing mean metabolite concentrations (*n*=3 per group). The axis legend unit is the *Z* score, and the data shown in Fig. 5A are utilized to generate this heatmap

Next, we employed LC-MS/MS to measure metabolite concentrations in tumor interstitial fluid. We observed a decrease in concentrations of glucose, glutamine, leucine, phenylalanine, and proline in the YUMMER1.7 TME (Fig. 4A, B). In contrast, the concentrations of lactate and lysine were significantly higher in the YUMMER1.7 TME.

YUMMER1.7 tumors utilize more glucose and glutamine than YUMM1.7 tumors in vivo

**Fig. 5.**
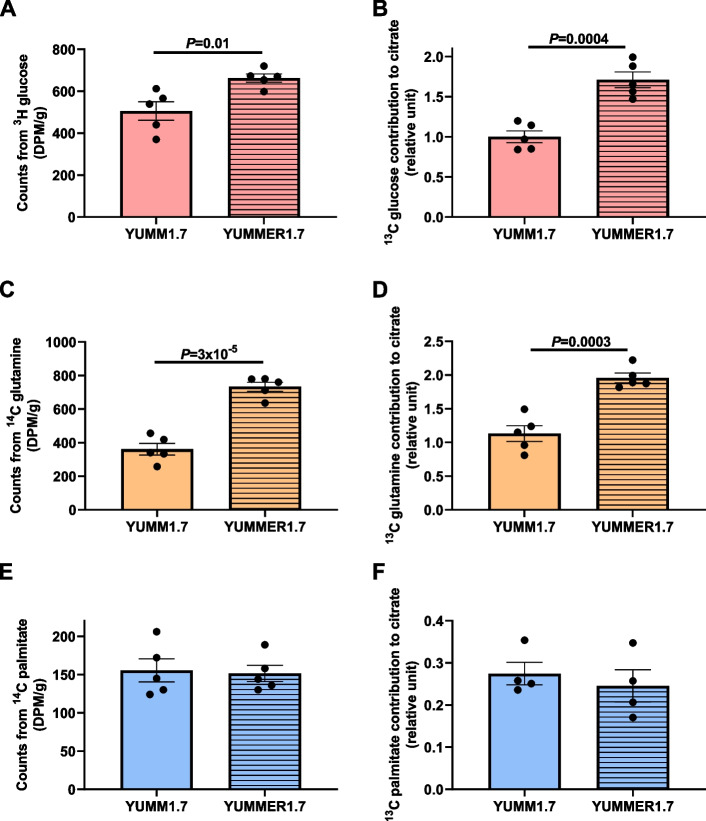
YUMMER1.7 tumors utilize more glucose and glutamine than YUMM1.7 tumors in vivo. **A**, **B** Tumor glucose uptake and oxidation. **C**, **D** Glutamine uptake and oxidation. **E**, **F** Palmitate uptake and oxidation. In all panels, groups were compared by the 2-tailed unpaired Student’s *t* test

We performed steady-state (Supplementary Figure S3A-C) stable and radioisotope infusions to study substrate uptake in melanoma tumors in awake mice in vivo. Our data demonstrate that despite showing no differences in size, YUMMER1.7 tumors had higher uptake of glucose (Fig. 5A) and glutamine (Fig. 5C), while palmitate uptake remained the same between the two melanoma models (Fig. 5E). We used stable isotope infusions to examine the relative contributions of glucose, glutamine, and palmitate to V_CS_ in tumor tissues and observed a similar result to the in vitro study. Glucose (Fig. 5B) and glutamine (Fig. 5D) had higher oxidation rates in YUMMER1.7 relative to YUMM1.7 tumors, whereas palmitic acid oxidation did not significantly differ between the two models (Fig. 5F).

### Glutamine transporter expression is a prognostic factor in melanoma patients

**Fig. 6:**
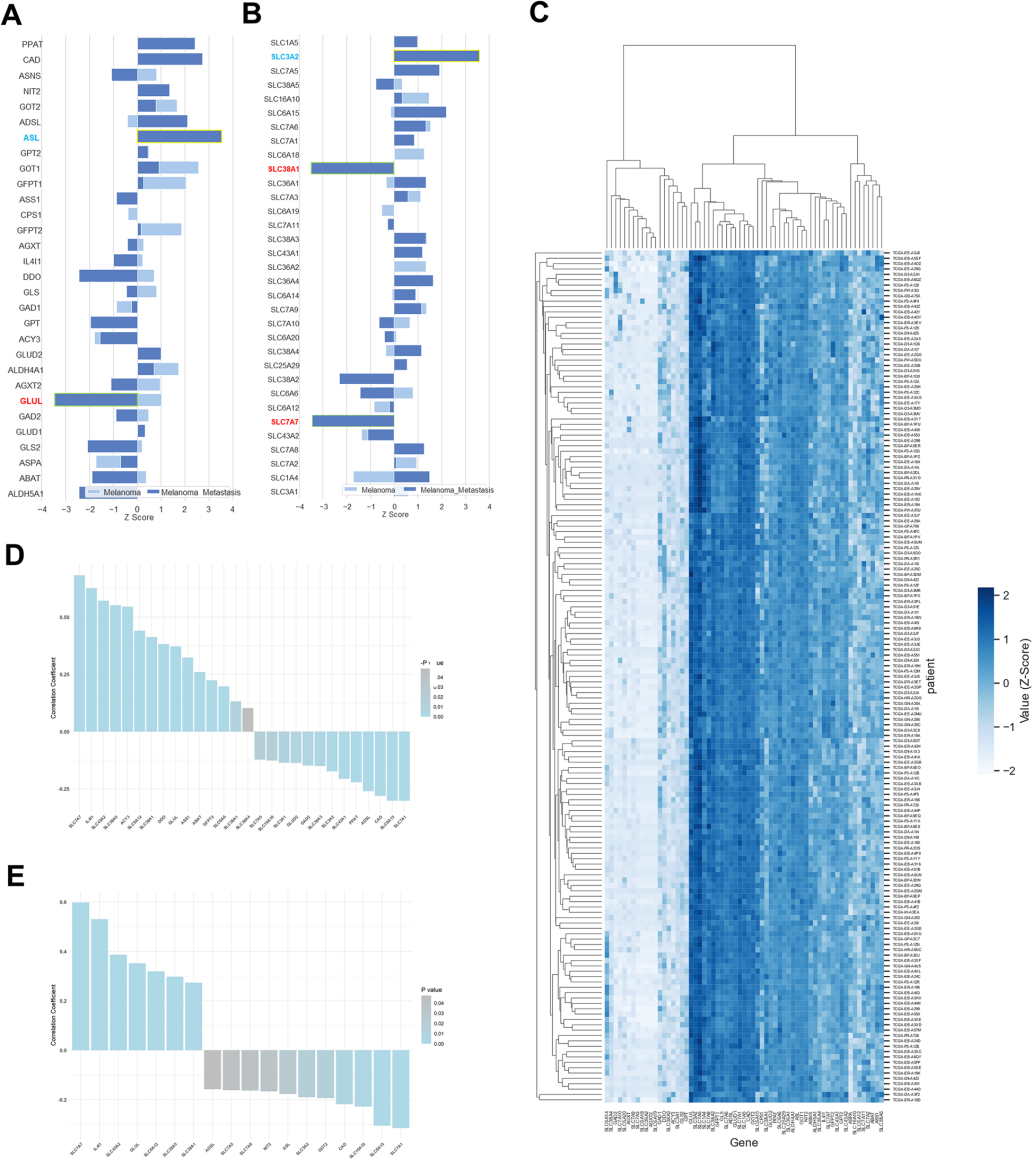
Amino acid transporters and metabolic enzymes are associated with beneficial outcomes in melanoma patients. *Z* scores of survival effect due to mRNA expression of amino acid metabolic enzymes (**A**) and transporters (**B**) in melanoma patients from PRECOG database. **C** Clustering heatmap of amino acid transporter and metabolic enzyme gene expression in 159 melanoma patients from TCGA database. The results shown here are entirely or partly based upon data generated by the TCGA Research Network: https://www.cancer.gov/tcga. **D** Correlation coefficient (*r* value) of leukocyte infiltration to amino acid metabolic enzymes and transporter mRNA expression levels in TCGA melanoma patients. *P*< 0.05. **E** Correlation coefficient (*r* value) of cytolytic activity to amino acid metabolic enzymes and transporter expression levels in TCGA melanoma patients. *P*< 0.05

Finally, we examined the prognostic impact of glutamine metabolism in melanoma patients, utilizing melanoma tumor gene expression from the PRECOG database [35]. A positive *Z* score indicates a detrimental effect on overall survival, and a negative *Z* score indicates improved survival, with (−)3.09 correlating to a *p* value of 0.001, which we deemed as significant as described previously [35]. Glucose and fatty acid metabolic genes mostly had a detrimental effect in both early- and late-stage melanoma patients (Supplementary Figure S4A and S4B). By contrast, higher expression of metabolic genes, as well as *GLUL*, correlated with an improved effect on prognosis, especially for late-stage melanoma patients with metastasis (Fig. 6A). Furthermore, although high expression of most amino acid transporters was correlated with increased mortality, elevated expression of *SLC38A1* (glutamine transporter) and *SLC7A7* (lysine transporter) had strong benefits for patients with metastatic melanoma (Fig. 6B) [35]. Though there was no obvious subsetting of melanoma patients by automated clustering, there were distinct amino acid gene clusters that varied in high vs. low expression across all melanoma patients (Fig. 6C).

Although our study is primarily focused on the impact of tumor metabolism on tumor-immune cell metabolic crosstalk and outcomes, we also examined the connection between tumor metabolism and anti-tumor lymphocyte infiltration and function. We calculated the correlation coefficient of leukocyte fraction (identified from pathology images using deep learning [36]) to amino acid metabolism-related gene expression in 369 melanoma patients from the TCGA database (Supplementary Figure S5B). We identified 14 genes (*SLC7A7, IL41, SLC43A2, SLC38A5, ACY3, SLC6A12, SLC38A1, DDO, GLUL, ASS1, ABAT, GFPT2, SLC6A6, SLC36A1, SLC38A4*) that correlate positively and 13 genes (*SLC7A5, SLC16A10, SLC3A1, GLUD2, GAD2, SLC3A2, SLC43A1, PPAT, ADSL, CAD, SLC6A15, SLC7A1*) that correlate negatively with leukocyte fraction in tumor tissue (Fig. 6D), with similar patterns observed with gene expression correlations with cytolytic activity (Fig. 6E). In contrast, we found that patients’ overall survival time did not correlate with leukocyte fraction within tumor tissue (Supplementary Figure S5A), implying that other factors, including anti-tumor function of lymphocytes, are important than strict lymphocyte fraction for clinical outcomes.

We approximated this lymphocyte effector function by estimating immune cytolytic activity (CYT). CYT is the geometric mean of the expression level of *GZMA*—which encodes serine protease—and *PRF1*—which encodes perforin. GZMA and PRF1 are the key enzymes involved in cytotoxic T lymphocyte effector function [37]. Higher CYT indicates greater cytotoxic T cell activity. By studying 159 patients from the TCGA database (Supplementary Figure S3C), we identified 10 genes (*SLC7A7, IL41, SLC43A2, GLUL, SLC6A12, SLC38A5, SLC38A1, DDO, SLC38A4*) that correlate positively and 11 genes (*ADSL, SLC7A5, NIT2, ASL, SLC38A2, GOT2, CAD, SLC16A10, SLC6A15, SLC7A1*) that correlate negatively with CYT (Fig. 5E).

## Discussion

Substrate preference is a teleological concept based on correlations between nutrient utilization and outcomes, rather than a single measurable variable. For this reason, we take a “multi-omics” approach to define how differences in metabolism may correlate with tumor immunogenicity in melanoma cells. We combine several analyses, including gene expression, metabolic flux, and survival in environments with restricted substrate availability. We use these methods to examine how the immunologically “hot” or “cold” identity of melanoma tumors reciprocally affects tumor cell substrate preference within the TME.

Researchers have long considered glutamine an essential nutrient for lymphocyte proliferation and effector function [38, 39], although a recent study on melanoma-bearing mice paradoxically suggested that glutamine deprivation enhances anti-tumor function and responsiveness to anti-PD1 treatment [21]. Most of these studies, however, were conducted in an in vitro T cell culture system and lack in vivo evidence to confirm the physiologic impact of T cell metabolism on tumor cells in the TME. Our data demonstrate that glutamine is a key substrate for tumor cells themselves in immunologically “hot” melanoma, both in vivo and in vitro*.*

Our study reveals divergent roles for glutamine in the TME between two genetically similar, immunologically “cold” (YUMM1.7) and “hot” (YUMMER1.7) murine tumor models. We compared RNA expression between YUMM1.7 and YUMMER1.7 to avoid confounding by the inclusion of TIL gene expression. We found that genes belonging to glucose and amino acid metabolism pathways are enriched in YUMMER1.7 compared to YUMM1.7. We then used isotope tracing methods to compare metabolic fluxes in both in vitro cell culture and in vivo tumor tissue. We discover that the uptake of glutamine and glucose, as well as the relative oxidation of both glucose and glutamine, is higher in YUMMER1.7 than in YUMM1.7. These data are consistent with our RNA-seq analysis, which indicated that glucose and glutamine metabolism pathways are enriched in YUMMER1.7. The uptake and oxidation rate of palmitic acid, by contrast, was similar between the two models.

We recognize that there are potential limitations to any tracer method. These limitations include (1) the invalidity of data when intracellular tracer enrichment and concentration are not at steady-state, (2) the possibility of entry of unaccounted anaplerotic substrates or metabolic pathways (for example, dilution of [^13^C] acetyl CoA by unlabeled citrate through ATP citrate lyase), and (3) incomplete equilibration between cytosolic and mitochondrial substrate pools. Additionally, we cannot rule out a contribution of altered metabolism in other cell populations within the tumor, such as immune and stromal cells, particularly if the fraction of these cell types differs between YUMM1.7 and YUMMER1.7 tumors. However, the results of our in vitro studies demonstrating differing effects of glucose and glutamine in melanoma cell lines with varying immunogenicity indicate that there are cell-specific effects of metabolic changes in melanoma. Altogether, we suggest that studies should use isotope tracer methods in combination with independent functional readouts, such as the in vitro substrate incubation studies included herein.

We also measured the concentrations of metabolites in tumor interstitial fluid. We identified a decrease in key nutrients, including glucose, alanine, proline, phenylalanine, and glutamine, in the YUMMER1.7 TME. These data indicate that a smaller quantity of key nutrients are available to CD8+ T cells in the immunotherapy-responding melanoma model (that is, in YUMMER1.7, the “hot” tumor model). Considering the increased anaplerotic flux in YUMMER1.7 cells, the lower substrate concentrations in the YUMMER1.7 TME likely represent increased substrate uptake. This highlights the importance of using tracers to study metabolism, rather than simply relying on nutrient concentrations.

In contrast, the concentration of lysine was higher in the YUMMER1.7 TME. Although lysine is an essential amino acid that enters the TCA cycle [40], the relationship between lysine availability and immune cell function is not well understood. Further work is necessary to explore whether therapies seeking to improve melanoma immunotherapy efficacy should target lysine metabolism.

We also found an increase in the lactate concentration in the YUMM1.7 TME. This might be due to increased rates of glycolysis in YUMM1.7. Glycolysis is elevated in YUMM1.7 to compensate for the relative decrease in anaplerotic substrate supply and, consequently, the expected decrease in oxidative phosphorylation.

Finally, and most importantly with regard to clinical implications, we studied the impact of amino acid uptake and amino acid metabolism on human melanoma patients. Unlike elevated glucose and fatty acid metabolic gene expression, which is generally deleterious for patients, heightened anaplerotic gene expression (e.g., D-aspartate oxidase (*DDO*) or glutaminase 2 (*GLS2*)) is a prognostic indicator of a beneficial effect. This effect is especially apparent in late-stage melanoma patients. We also used the TCGA database to study the impact of amino acid metabolism gene expression on TIL infiltration and cytolytic activity (CYT) in melanoma patients. We found that glutamine transporter expression in tumor tissue is positively correlated with both leukocyte infiltration and CYT. Based on our and others’ data demonstrating that tumor cells actively take up and oxidize glutamine, tumor tissue with higher glutamine transporter expression have a higher capacity to “soak up” glutamine, leading to glutamine deprivation in the TME. This again suggests that lower glutamine concentration in the TME is beneficial for CD8+ T cell anti-tumor function.

This study has the inevitable caveat that human RNA-seq data is obtained from tumor samples that may or may not contain TILs. Thus, one can argue the positive association between anaplerotic gene expression and lymphocyte activation is due to increased activated lymphocytes in sequencing tissue rather than due to increased anaplerotic gene expression in tumor tissue itself. Regardless, our results validate anaplerotic gene expression as a prognostic factor for lymphocyte effector function and potentially predict responsiveness to immune checkpoint inhibitors.

While our study lays a foundation for further studies of competition for substrates between tumor and immune cells in the TME in “immunologically hot” melanoma, we do not test competition with immune cells. Further experiments will be needed to directly test the “substrate competition” hypothesis. These studies must also understand that the idea of substrate competition by definition is teleological and will be almost impossible to conclusively prove experimentally. In other words, we can measure substrate uptake and use, but without the ability to interview tumor cells and immune cells, it is difficult to prove which substrate the cells “seek.” Even so, the use of isotope tracers enables a unique window into substrate utilization in tumor cells in vivo and in vitro.

## Conclusions

In sum, our study provides insight into how different tumor subtypes vary in metabolic flexibility and substrate preference, suggesting that human tumor subtypes, too, may vary along such dimensions. Unlike many prior studies, the focus of the current work is on how tumor metabolism predicts immunogenicity, as opposed to primarily centering immunometabolism. These differences in metabolism between immunologically hot and cold melanoma cell lines may be exploited to design metabolic therapies to enhance immunotherapy efficacy and overcome immunotherapy resistance in patients with melanoma and potentially other cancers.

## Supplementary Information

**Additional file 1:** **Supplementary Table S1.** Differentially expressed genes comparing YUMMER1.7 to YUMM1.7. This 650 page table is uploaded as supplementary material exclusively, due to its length.

**Additional file 2: Supplementary Figure S1.** Mass isotopomer distribution in YUMM1.7 and YUMMER1.7 cells incubated in [U-^13^C6] glucose, [U-^13^C_5_] glutamine, or [U-^13^C_16_] palmitate. *P<0.05, **P<0.01, ***P<0.001, ****P<0.0001 by the 2-tailed unpaired Student’s t-test.

**Additional file 3: Supplementary Figure S2.** YUMMER1.7 cells exhibit more anaplerosis *in vitro* than YUMM1.7 cells. (A)-(E) ^13^C enrichment of TCA cycle intermediates in cells incubated in [U-^13^C_6_] glucose. (F)-(J) ^13^C enrichment of TCA cycle intermediates in cells incubated in [U-^13^C_5_] glutamine. V_CS_ from [U-^13^C_5_] glutamine. (F) V_CS_ from [U-^13^C_16_] palmitate. In panels (E) and (F), data were normalized to V_CS_ data from YUMM1.7 cells incubated in the same tracer. (F) V_CS_ from [U-^13^C_16_] palmitate. In all panels, data were compared by the 2-tailed unpaired Student’s t-test.

**Additional file 4:** **Supplementary Figure S3.** Mice reach steady-state in the contribution of precursors to citrate within 60 minutes. (A) [U-^13^C_6_] glucose, (B) [U-^13^C_5_] glutamine, and (C) [U-^13^C_16_] palmitate tracers were used. The solid lines denote the mean of n=5 per cell line. No significant differences between 60 and 150 min were observed using the 2-tailed unpaired Student’s t-test.

**Additional file 5:** **Supplementary Figure S4.** PRECOG data of glucose and fatty acid metabolic enzymes in melanoma patients. (A) Z scores of survival effect due to mRNA expression of glucose metabolic enzymes in melanoma patients from PRECOG database. (B) Z scores of survival effect due to mRNA expression of fatty acid metabolic enzymes in melanoma patients from PRECOG database.

**Additional file 6:** **Supplementary Figure S5.** Analysis of RNA seq data in melanoma patients from TCGA database. (A) Dot plot of overall survival time and leukocyte infiltration fraction in 369 melanoma patients. Adjusted R^2^=-0.001507, *P* value=0.5046. (B) Correlation matrix of leukocyte fraction to amino acid metabolic enzyme and transporter gene expression in 369 melanoma patients from TCGA database. (C) Correlation matrix of cytolytic activity to amino acid metabolic enzyme and transporter gene expression in 159 melanoma patients from TCGA database.

## Data Availability

The datasets used and/or analyzed during the current study are available from the corresponding author on reasonable request.

## Abbreviations

CBI: Checkpoint-Blockade Immunotherapy
CYT: Cytolytic activity
DCA: Dichloroacetate
NEFA: Non-esterified fatty acid
PCA: Principal Component Analysis
TCA: TriCarboxylic acid cycle
TIL: Tumor-infiltrating lymphocyte
TME: Tumor microenvironment
