# Supplementary material for: Isotope tracing reveals distinct substrate preference in murine melanoma subtypes with differing anti-tumor immunity
Source: Cancer Metab. 2022 Dec 1;10:21. doi: 10.1186/s40170-022-00296-7 (PMC9714036; doi:10.1186/s40170-022-00296-7)
Supplement: Supplementary file 2 — Additional file 2: Supplementary Figure S1. Mass isotopomer distribution in YUMM1.7 and YUMMER1.7 cells incubated in [U-13C6] glucose, [U-13C5] glutamine, or [U-13C16] palmitate. *P<0.05, **P<0.01, ***P<0.001, ****P<0.0001 by the 2-tailed unpaired Student’s t-test. [file 40170_2022_296_MOESM2_ESM.docx]

**Supplementary Figure S1. Mass isotopomer distribution in YUMM1.7 and YUMMER1.7 cells incubated in [U-^13^C6] glucose, [U-^13^C_5_] glutamine, or [U-^13^C_16_] palmitate.** **P*<0.05, ***P*<0.01, ****P*<0.001, *****P*<0.0001 by the 2-tailed unpaired Student’s t-test.
