# Supplementary material for: Isotope tracing reveals distinct substrate preference in murine melanoma subtypes with differing anti-tumor immunity
Source: Cancer Metab. 2022 Dec 1;10:21. doi: 10.1186/s40170-022-00296-7 (PMC9714036; doi:10.1186/s40170-022-00296-7)
Supplement: Supplementary file 3 — Additional file 3: Supplementary Figure S2. YUMMER1.7 cells exhibit more anaplerosis in vitro than YUMM1.7 cells. (A)-(E) 13C enrichment of TCA cycle intermediates in cells incubated in [U-13C6] glucose. (F)-(J) 13C enrichment of TCA cycle intermediates in cells incubated in [U-13C5] glutamine. VCS from [U-13C5] glutamine. (F) VCS from [U-13C16] palmitate. In panels (E) and (F), data were normalized to VCS data from YUMM1.7 cells incubated in the same tracer. (F) VCS from [U-13C16] palmitate. In all panels, data were compared by the 2-tailed unpaired Student’s t-test. [file 40170_2022_296_MOESM3_ESM.docx]

**Supplementary Figure S2. YUMMER1.7 cells exhibit more anaplerosis *in vitro* than YUMM1.7 cells.** (A)-(E) ^13^C enrichment of TCA cycle intermediates in cells incubated in [U-^13^C_6_] glucose. (F)-(J) ^13^C enrichment of TCA cycle intermediates in cells incubated in [U-^13^C_5_] glutamine. V_CS_ from [U-^13^C_5_] glutamine. (F) V_CS_ from [U-^13^C_16_] palmitate. In panels (E) and (F), data were normalized to V_CS_ data from YUMM1.7 cells incubated in the same tracer. (F) V_CS_ from [U-^13^C_16_] palmitate. In all panels, data were compared by the 2-tailed unpaired Student’s t-test.
