# Supplementary material for: Isotope tracing reveals distinct substrate preference in murine melanoma subtypes with differing anti-tumor immunity
Source: Cancer Metab. 2022 Dec 1;10:21. doi: 10.1186/s40170-022-00296-7 (PMC9714036; doi:10.1186/s40170-022-00296-7)
Supplement: Supplementary file 4 — Additional file 4: Supplementary Figure S3. Mice reach steady-state in the contribution of precursors to citrate within 60 minutes. (A) [U-13C6] glucose, (B) [U-13C5] glutamine, and (C) [U-13C16] palmitate tracers were used. The solid lines denote the mean of n=5 per cell line. No significant differences between 60 and 150 min were observed using the 2-tailed unpaired Student’s t-test. [file 40170_2022_296_MOESM4_ESM.docx]

**Supplementary Figure S3. Mice reach steady-state in the contribution of precursors to citrate within 60 minutes.** (A) [U-^13^C_6_] glucose, (B) [U-^13^C_5_] glutamine, and (C) [U-^13^C_16_] palmitate tracers were used. The solid lines denote the mean of n=5 per cell line. No significant differences between 60 and 150 min were observed using the 2-tailed unpaired Student’s t-test.
