# Supplementary material for: Isotope tracing reveals distinct substrate preference in murine melanoma subtypes with differing anti-tumor immunity
Source: Cancer Metab. 2022 Dec 1;10:21. doi: 10.1186/s40170-022-00296-7 (PMC9714036; doi:10.1186/s40170-022-00296-7)
Supplement: Supplementary file 5 — Additional file 5: Supplementary Figure S4. PRECOG data of glucose and fatty acid metabolic enzymes in melanoma patients. (A) Z scores of survival effect due to mRNA expression of glucose metabolic enzymes in melanoma patients from PRECOG database. (B) Z scores of survival effect due to mRNA expression of fatty acid metabolic enzymes in melanoma patients from PRECOG database. [file 40170_2022_296_MOESM5_ESM.docx]

**
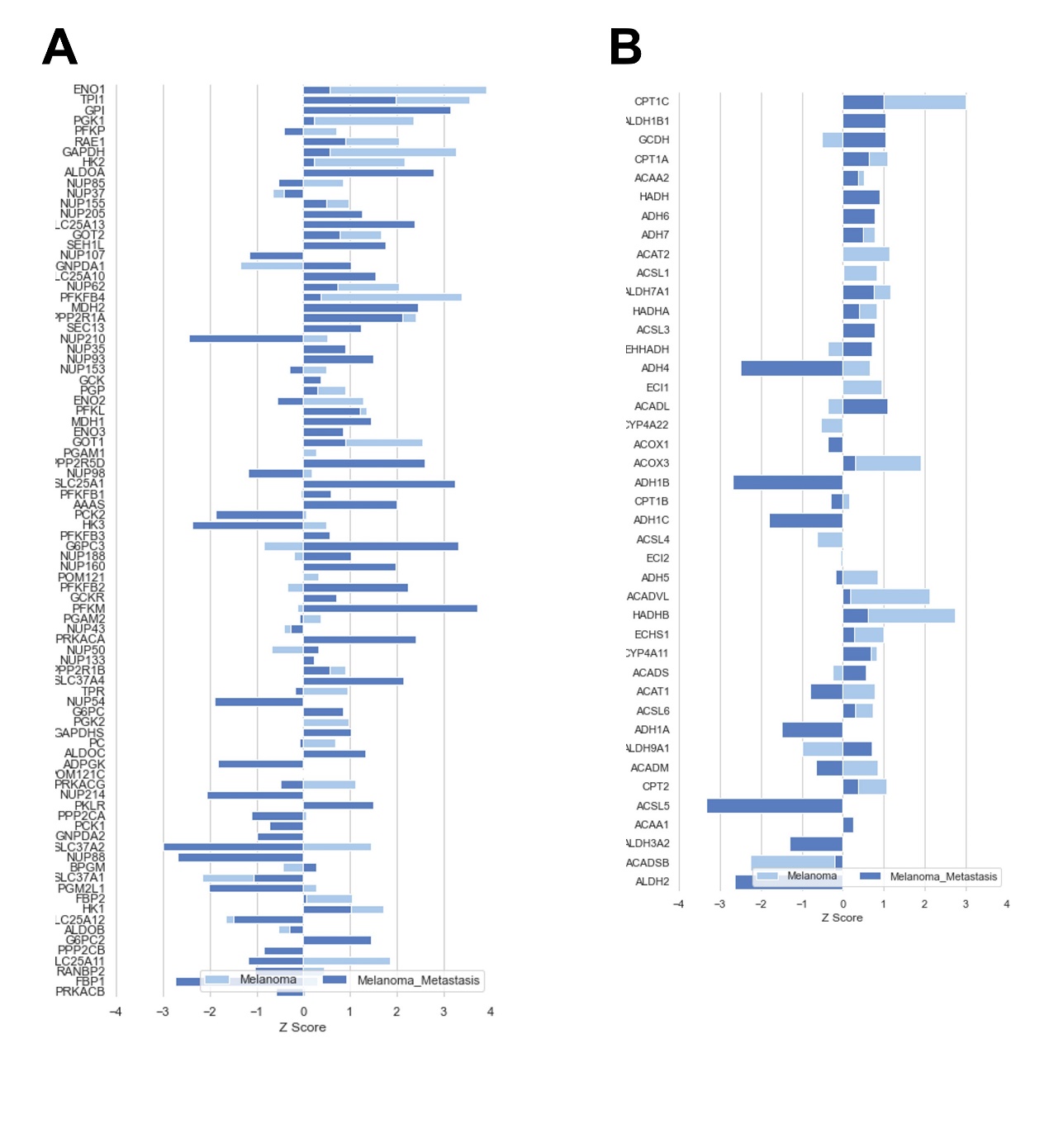
**

**Supplementary Figure S4. PRECOG data of glucose and fatty acid metabolic enzymes in melanoma patients.** (A) Z scores of survival effect due to mRNA expression of glucose metabolic enzymes in melanoma patients from PRECOG database. (B) Z scores of survival effect due to mRNA expression of fatty acid metabolic enzymes in melanoma patients from PRECOG database.
