# Supplementary material for: Isotope tracing reveals distinct substrate preference in murine melanoma subtypes with differing anti-tumor immunity
Source: Cancer Metab. 2022 Dec 1;10:21. doi: 10.1186/s40170-022-00296-7 (PMC9714036; doi:10.1186/s40170-022-00296-7)
Supplement: Supplementary file 6 — Additional file 6: Supplementary Figure S5. Analysis of RNA seq data in melanoma patients from TCGA database. (A) Dot plot of overall survival time and leukocyte infiltration fraction in 369 melanoma patients. Adjusted R2=-0.001507, P value=0.5046. (B) Correlation matrix of leukocyte fraction to amino acid metabolic enzyme and transporter gene expression in 369 melanoma patients from TCGA database. (C) Correlation matrix of cytolytic activity to amino acid metabolic enzyme and transporter gene expression in 159 melanoma patients from TCGA database. [file 40170_2022_296_MOESM6_ESM.docx]

**
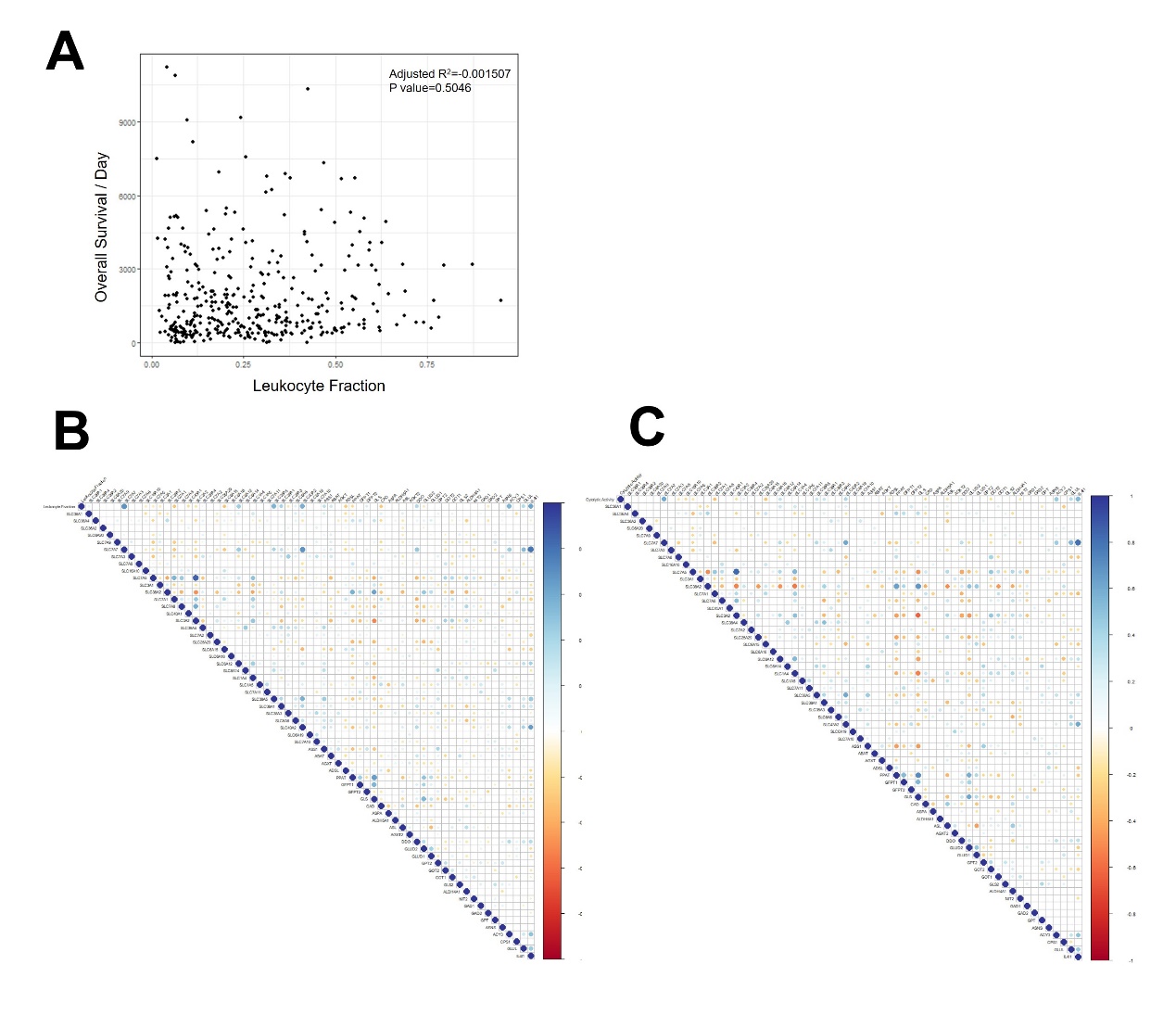
 Supplementary Figure S5. Analysis of RNA seq data in melanoma patients from TCGA database.** (A) Dot plot of overall survival time and leukocyte infiltration fraction in 369 melanoma patients. Adjusted R^2^=-0.001507, P value=0.5046. (B) Correlation matrix of leukocyte fraction to amino acid metabolic enzyme and transporter gene expression in 369 melanoma patients from TCGA database. (C) Correlation matrix of cytolytic activity to amino acid metabolic enzyme and transporter gene expression in 159 melanoma patients from TCGA database.
